# Rat Chondrocyte-Associated Antigen Identified as Sialylated Transmembrane Protein Tmp21 Belonging to the p24 Protein Family

**DOI:** 10.1007/s00223-013-9816-5

**Published:** 2013-11-23

**Authors:** Anna Osiecka-Iwan, Justyna Niderla-Bielinska, Anna Hyc, Stanislaw Moskalewski

**Affiliations:** Department of Histology and Embryology, Medical University of Warsaw, Chalubińskiego 5, 02004 Warsaw, Poland

**Keywords:** Chondrocyte-associated antigen, Tmp21, p24 protein, Rat

## Abstract

Rabbit serum produced after transplantation of isolated rat chondrocytes [sensitized rabbit serum (SRS)] demonstrated *M*_r_ ~ 74- and ~23-kDa (western blot analysis) antigens in rat chondrocyte extracts. Only the latter remained after reduction in 2-mercaptoethanol. Protein sequence analysis of 23-kDa chondrocyte-associated antigen (CAA) revealed that it corresponds to transmembrane Tmp21 protein belonging to the p24 protein family. These proteins mainly participate in the traffic between the endoplasmic reticulum and Golgi complex and in some cells appear also in the membrane of secretory granules and plasmalemma. Tmp21 extracted from chondrocytes was sialylated and ceased to bind SRS after deglycosylation. A previous study from our laboratory indicated that expression of CAA, now identified as sialylated Tmp21, decreased in cultured chondrocytes concomitantly with the decline of collagen type II and aggrecan and the rise of collagen type I and versican expression. Since the sialylated form of Tmp21 (also known as emp24) was not described in other tissues and seems to be specific for chondrocytes, we assume that CAA may be considered a chondrocyte differentiation antigen.

## Introduction

In the course of studies on the xenogeneic transplantation of chondrocytes we have found that serum of rabbits sensitized by transplants of rat chondrocytes [sensitized rabbit serum (SRS)] contained antichondrocyte cytotoxic antibodies. This serum reacted also in western blot with *M*_r_ ~ 74- and ~23-kDa antigens present in chondrocyte lysates. Only the latter remained after reduction in 2-mercaptoethanol. Neither antigen could be detected by this serum in lysates of fibroblasts, epitheliocytes, or thymocytes [1]. Thus, we have suggested that the produced serum reacted with chondrocyte-associated antigen (CAA), but the nature of the antigen remained unknown. In the present work proteins extracted from articular–epiphyseal chondrocytes were sequenced and the protein corresponding to the ~23-kDa antigen was identified as transmembrane Tmp21 protein belonging to the p24 protein family. These proteins mainly participate in the traffic between the endoplasmic reticulum (ER) and Golgi complex but in some cells appear also in membranes of secretory vacuoles [2] and on cell surfaces [3, 4]. We have found that Tmp21 (also termed p23, p24δ, transmembrane emp24-like trafficking protein 10 or TMED10 in the protein database; see Strating et al. [5]) extracted from chondrocytes was sialylated and ceased to bind SRS after deglycosylation. Since, as far as we could establish, the sialylated form of Tmp21 has not been described in other tissues, Tmp21 with its sialylated oligosaccharide moiety could have some tissue specificity and, thus, qualify as a chondrocyte differentiation antigen.

## Materials and Methods

### Preparation of Cartilage and Detergent Extraction of Chondrocyte Surface Macromolecules

Inbred Lewis rats, 3–5 days old, obtained from the Animal Unit of the Medical University of Warsaw, served as donors of cartilage. The study was approved by the Animal Ethics Committee of the Medical University of Warsaw. Chondrocytes were isolated from articular–epiphyseal complexes, cartilaginous fragments of ribs, nasal septa, and tracheal cartilaginous rings. All types of cartilage were carefully cleaned from the surrounding tissues and digested using 0.25 % collagenase, 0.05 % DNase, and 7 nM *N*-α-tosyl-l-lysyl chloromethyl ketone (all from Sigma-Aldrich, Diesenhofen, Germany) at 37 °C for 3 h. When the whole cartilage was dissolved, the cells were filtered through 40-μm mesh nylon cloth (Millipore, Bedford, MA), washed three times in RPMI (Sigma-Aldrich), and seeded into 35-mm Petri dishes (5 × 10^6^ cells per dish; Corning, Corning, NY) in 2 mL of serum-free RPMI. After 24 h, surface macromolecules were extracted with 50 mM Bis–Tris/HCl buffer (pH 7) containing 750 mM aminocaproic acid and 125 μL of 10 % laurylmaltoside (all from Sigma-Aldrich) per 1 mL of buffer as described previously [6]. The extract was desalted on PD-10 columns (Amersham Biosciences, Uppsala, Sweden), lyophilized, and dissolved in distilled water.

### Protein Determination

One microliter of chondrocyte extract or extraction buffer (blank test) and 9 μL of deionized water were placed in a flat-bottomed 96-well plate (Corning), and 0.2 mL of BCA protein assay reagent (Pierce, Rockford, IL) was added to each well. The plate was incubated at 37 °C for 30 min. Protein concentration was determined spectrophotometrically at 550 nm (SLT Spectra Lab Instruments, Crailsheim, Germany).

### Electrophoresis and Western Blotting

Chondrocyte surface macromolecules (10 μg of protein) mixed in sample buffer with or without mercaptoethanol (Sigma-Aldrich) were separated by sodium dodecyl sulfate-polyacrylamide gel electrophoresis, SDS-PAGE (12 % acrylamide; Bio-Rad Laboratories, Hercules, CA). Separated proteins were transferred onto a PVDF membrane (Bio-Rad) by semidry blotting at 25 V for 30 min using the Trans-Blot SD apparatus. SRS diluted 1:120 served as the primary antibody. Incubation lasted for 1 h. The serum was prepared in rabbits (New Zealand white rabbits, obtained from the Institute of Zootechnology in Cracow, Poland) injected intramuscularly three times with 5 × 10^7^ of living chondrocytes derived from the articular–epiphyseal cartilage complexes of 3–5-day-old rats at 2-week intervals [1]. Biotinylated F(ab′)_2_ fragments of swine anti-rabbit immunoglobulins (Dako, Glostrup, Denmark) served as the secondary antibody. Antibody binding was demonstrated by an amplified alkaline phosphatase detection system (Bio-Rad). Anti-Tmp21 (Thermo Scientific, Pierce Biotechnology, Rockford, IL) and anti-TMED7 antibody (Abcam, Cambridge, UK), both polyclonal and produced in rabbits, were used with the same detection system. The relative molecular weight of the antigens was calculated using GelWorks 1D Intermediate software (Bio-Rad) in relation to the prestained SDS-PAGE (low range) standard (Bio-Rad). The second polyacrylamide gel after protein electrophoresis was stained with Coomassie blue, and the band corresponding to the band detected by SRS was cut for protein sequence analysis.

### Deglycosylation of Extracted Macromolecules

Surface macromolecules extracted from articular–epiphyseal chondrocytes (30 μg of protein) were deglycosylated using the Enzymatic Protein Deglycosylation Kit (Sigma-Aldrich) according to the manufacturer’s protocol. Briefly, protein samples were incubated with endo-*O*-glycosidase, β-1,4-galactosidase, β-*N*-acetylglucosaminidase, PNGase F, or α-2(3,6,8,9)-neuraminidase (sialidase A). After deglycosylation, protein samples (10 μg of protein) were mixed in sample buffer and separated by SDS-PAGE.

### Protein Sequence Analysis

Analysis and data processing were done on a commercial basis in the Mass Spectrometry Laboratory, Institute of Biochemistry and Biophysics of the Polish Academy of Sciences, Warsaw, Poland, according to the following procedures:

#### Trypsin Digestion

Gel slices were subjected to a standard “in-gel digestion” procedure during which proteins were reduced with 100 mM 1,4-dithiothreitol (Sigma-Aldrich) (for 30 min at 56 °C), alkylated with iodoacetamide (45 min in a dark room at room temperature), and digested overnight with trypsin (sequencing grade modified trypsin, V5111; Promega, Madison, WI). Resulting peptides were eluted from gel with 0.1 % trifluoroacetic acid (TFA) and 2 % acetonitrile, ACN (Sigma-Aldrich).

#### Mass Spectrometry

Peptide mixtures were separated by liquid chromatography prior to molecular mass measurements on an Orbitrap Velos mass spectrometer (Thermo Electron, San Jose, CA). The peptide mixture was applied to an RP-18 precolumn (nanoACQUITY Symmetry^®^ C18; Waters, Milford, MA) using water containing 0.1 % TFA as mobile phase and then transferred to a nano-HPLC RP-18 column (nanoACQUITY BEH C18, Waters) using an ACN gradient (0–60 % ACN in 70 min) in the presence of 0.05 % formic acid with a flow rate of 150 nL/min. The column outlet was directly coupled to the ion source of the spectrometer working in the regime of data-dependent MS to MS/MS switch. A blank run ensuring lack of cross-contamination from previous samples preceded each analysis.

#### Data Processing

Raw data were processed by a Mascot Distiller followed by a database search with the Mascot program (Matrix Science, London, UK; 8-processor on-site license) against NCBInr (version 20100203). Search parameters for precursor and product ion mass tolerance were 40 ppm and 0.8 Da, respectively, with allowance made for one missed trypsin cleavage and the following fixed modifications: cysteine carbamidomethylation and allowed variable modification, oxidation (M). Peptides with a Mascot score exceeding the threshold value corresponding to a <5 % false-positive rate, calculated by the Mascot procedure, were considered to be positively identified.

## Results

### Protein Sequence Determination

Mass spectrometric data processing of a protein present in the band reacting with SRS was repeated six times, and all determinations gave similar results. Results of one sequencing are given in Table 1. The studied protein had a mass of 22,289 Da and a Mascot score of 441 calculated on the basis of four matching peptides with an ion score in the range 50–138 (Table 1). Protein sequence coverage was 25 % and revealed as the best-matched protein the transmembrane protein Tmp21 (21-kDa transmembrane-trafficking protein). The BLAST search program classified it as a protein belonging to the p24 family.

**Table 1:** Amino acid sequences of peptides present in Tmp21 protein found by Mascot search

Numbers	Localization in protein (start–end)	Peptide sequences	Possible modifications
1	75–86	K. YTFAAHMDGTYK. F	Oxidation (M)
2	113–124	K. GQDMETEAHQNK. L	
3	125–140	K. LEEMINELAVAMTAVK. H	Oxidation (M)
4	141–149	K. HEQEYMEVR. E	Oxidation (M)

### Electrophoresis and Western Blotting

**Fig. 1:**
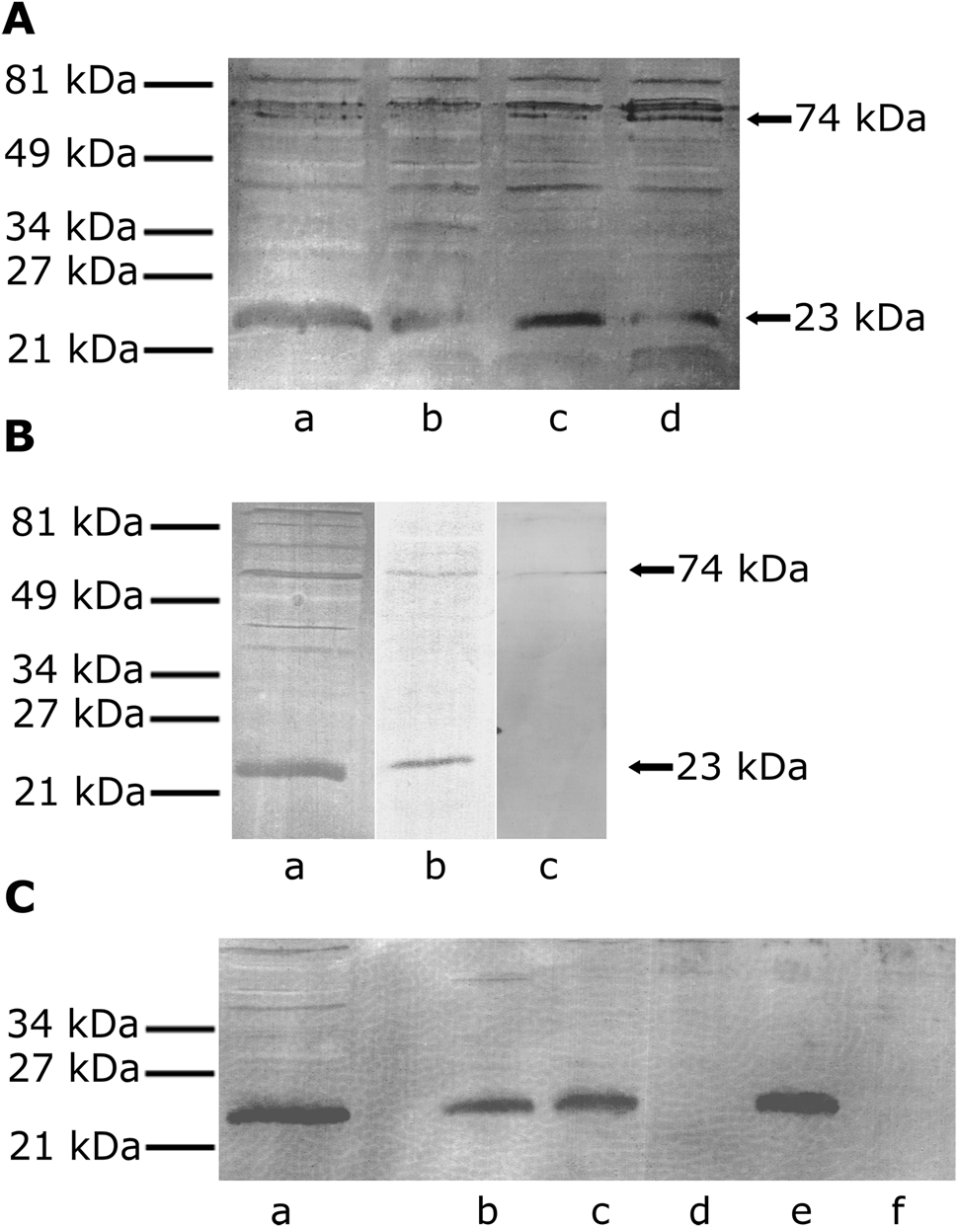
**a**–**c** SDS-PAGE and an immunoblot of chondrocyte extracts. As primary antibodies served as SRS, anti-Tmp21 or -TMED7 antibody, both polyclonal, was produced in rabbits. Biotinylated F(ab′)_2_ fragments of swine anti-rabbit immunoglobulins served as the secondary antibody. Antibody binding was demonstrated by an amplified alkaline phosphatase detection system. **a** (*a*) Articular–epiphyseal, (*b*) nasal, (*c*) rib, (*d*) tracheal chondrocytes. **b** Chondrocyte-associated antigen studied with (*a*) SRS, (*b*) polyclonal anti-Tmp21 antibody, (*c*) anti-TMED7 antibody. **c** (*a*) Control chondrocyte extract (without deglycosylation), (*b*–*f*) chondrocyte extracts digested with enzymes: (*b*) β-1,4-galactosidase, (*c*) endo-*O*-glycosidase, (*d*) α-2(3,6,8,9)-neuraminidase (sialidase A), (*e*) PNGase F, (*f*) β-*N*-acetylglucosaminidase. *Arrows* indicate* bands* corresponding to reduced mercaptoethanol (23 kDa) and nonreduced (74 kDa) antigen in which 23-kDa antigen was connected with presumably other members of the p24 family of proteins. The pattern of deglycosylation indicates that the Tmp21 protein is sialylated

SRS detected antigen with *M*_r_ of ~74 kDa in the nonreduced state and in the reduced state with *M*_r_ of ~23 kDa in articular–epiphyseal chondrocyte surface macromolecule extracts, as previously reported [1]. Antigens of similar molecular weight were also found among macromolecules extracted from nasal, tracheal, and rib chondrocytes and, judging from the width of electrophoretic bands, were particularly marked in the latter case (Fig. 1a).

Antibodies against Tmp21 detected antigen in articular–epiphyseal chondrocyte surface macromolecule extracts with *M*_r_ of ~74 kDa in the nonreduced state and in the reduced state with *M*_r_ of ~23 kDa (Fig. 1b). Antibodies against TMED7 protein (also termed gp27 or hp24γ_3_) did not react with chondrocyte extracts.

### Digestion with Deglycosylating Enzymes

Digestion of macromolecule samples with α-2(3,6,8,9)-neuraminidase (sialidase A), which cleaves the glycosidic linkages of neuraminic acids [7], or with β-*N*-acetylglucosaminidase, which catalyzes the hydrolysis of terminal, nonreducing β-*N*-acetylglucosamine residues from oligosaccharides [8], abolished antibody binding, while the remaining deglycosylating enzymes had no effect (Fig. 1c).

## Discussion

Proteins of the p24 family are involved in bidirectional transport at the ER–Golgi complex interface [9]. They are integral membrane proteins with a luminal domain of about 20 kDa, a single-span transmembrane domain, and a short C-terminal cytoplasmic tail of 12–18 amino acids [5, 10]. The cytoplasmic tail contains binding motifs for the vesicle coat complexes COPI and COPII [11]. At the luminal side, p24 proteins have an N-terminal Golgi dynamics (GOLD) domain, which may play a role in the incorporation of cargo into transport vesicles [9, 12]. The N-terminal luminal domain contains two conserved cysteine residues [13], presumably forming a disulfide bond within the GOLD domain [9]. In some p24 family members this domain is followed by a putative coiled-coil region that may interact with other p24 proteins forming larger, hetero-oligomeric complexes [4, 5]. According to Jenne et al. [14], however, individual members of the mammalian p24 family exist as heterodimers and monomers and the ratio between these two forms depends on both the investigated organelle (Golgi complex, ER) and the p24 protein.

On the basis of sequence homology, p24s were divided into four subfamilies: p24α, p24β, p24γ, and p24δ [15] or p23, p24, p25, and p26 [16, 17]. Full nomenclature of the p24 protein family is presented in a recent review [5]. Some members of the p24 protein family have been found outside the ER–Golgi compartment, in membranes of peroxisomes [18] or insulin granules [2]. The latter finding suggested that a fraction of p24 proteins enters post-Golgi compartments and, after fusion of granule and cell membranes, is exposed on the cell surface. Blum and Lepier [3] found that p23 (Tmp21) has a receptor-like luminal domain and a short cytoplasmic tail with an atypical ER retention KKXX motif. Despite the presence of this motif, p23 appears on the plasma membrane. When the KKXX motif is abolished, p23 shows extremely increased trafficking to the plasma membrane. Füllenkrug et al. [4] found that gp27 (hp24γ_3_, TMED7 in the protein database), another protein of the p24 family which cycles extensively in the early secretory pathway, is modified by enzymes of the Golgi apparatus with gradual conversion into a glycoprotein displaying complex and terminal oligosaccharides, the latter in the form of sialic acid. Moreover, gp27 coexpressed with p23 (Tmp21) migrated from the Golgi and was incorporated into the plasma membrane and lysosomes. Gp27 may also form a hetero-oligomeric complex with p23 (Tmp21), p24, and GMP25, which presumably exists in dynamic equilibrium; but the forces involved in complex formation are not well defined [19].

The CAA identified as Tmp21 in the nonreduced state formed complexes with *M*_r_ ~ 74. In view of the results presented by Füllenkrug et al. [4], it probably formed complexes with another member(s) of the p24 family of proteins. The reducing action of mercaptoethanol on this complex could probably be explained by disruption of disulfide bonds formed by two conserved cysteine residues present within the GOLD domain [5, 13] and destabilization of the complex. It is noteworthy that the expression of CAA gradually decreased in monolayer culture and was not detected beyond the 96th h. Expression of collagen type II and aggrecan significantly declined after 7 days of culture, dropped to a low level after 2 weeks, and was accompanied by a rise in collagen type I and versican expression [6]. The drop in collagen type II and aggrecan expression (evaluated by real-time PCR) occurred after a longer culture period than the disappearance of CAA observed in western blots. This phenomenon could be at least partially dependent on the sensitivity of the detection methods. Recent findings indicate that p24 proteins from the same subfamily are functionally nonredundant and have distinct functions in secretory cargo biosynthesis. In induced intermediary pituitary melanotropes of *Xenopus laevis*, a subset of p24 proteins was upregulated together with proopiomelanocortin (POMC), whereas two other p24 proteins were expressed but not coordinately with POMC [10, 20]. Thus, it is possible that collagen type II or aggrecan is transported in vesicles surrounded by membranes containing sialylated Tmp21, whereas either nonsialylated Tmp21 or another member of the p24 family participates in the transport of collagen type I or versican. Since CAA is present in chondrocytes from various organs, it is possible to hypothesize that Tmp21 represents a chondrocyte differentiation antigen involved in the transport of cartilage matrix proteins. It disappears after chondrocyte dedifferentiation and may be difficult to detect in chondrocytes which cease producing cartilage matrix.

Füllenkrug et al. [4] found that gp27 (TMED7) was sialylated in HeLa cells, whereas p23 (Tmp21) was not glycosylated. In our present work chondrocyte extracts did not contain protein reacting with anti-gp27 and Tmp21 was sialylated. We have previously found that anti-CAA serum lysed chondrocytes but not fibroblasts, endotheliocytes, or thymocytes. Furthermore, it was not detected with western blot in extracts from other cell types, i.e., fibroblasts, epitheliocytes, or thymocytes [1]. Thus, chondrocytes seem to be, as yet, the only recognized type of cells in which the sialylated form of a protein belonging to the p24 family acquires tissue specificity. Therefore, in view of tissue culture observations indicating loss of sialylated Tmp21 expression after the change of chondrocyte to a fibroblastic phenotype [6], its expression in all types of hyaline cartilage, and lack of expression in fibroblasts and endotheliocytes [1], we assume that it is a chondrocyte differentiation antigen.
